# Semi-Supervised Deep Learning in High-Speed Railway Track Detection Based on Distributed Fiber Acoustic Sensing

**DOI:** 10.3390/s22020413

**Published:** 2022-01-06

**Authors:** Shulun Wang, Feng Liu, Bin Liu

**Affiliations:** 1 Research Center of Network Management, Beijing Jiaotong University, Beijing 100044, China; fliu@bjtu.edu.cn; 2 Key Laboratory of Deep Oil and Gas, China University of Petroleum (East China), Qingdao 266580, China; b18010027@s.upc.edu.cn

**Keywords:** semi-supervised learning, CNN, track detection, deep learning, high-speed railway, DAS

## Abstract

High deployment costs, safety risks, and time delays restrict traditional track detection methods in high-speed railways. Therefore, approaches based on optical sensors have become the most remarkable strategy in terms of deployment cost and real-time performance. Owing to the large amount of data obtained by sensors, it has been proven that deep learning, as a powerful data-driven approach, can perform effectively in the field of track detection. However, it is difficult and expensive to obtain labeled data from railways during operation. In this study, we used a segment of a high-speed railway track as the experimental object and deployed a distributed optical fiber acoustic system (DAS). We propose a track detection method that innovatively leverages semi-supervised deep learning based on image recognition, with a particular pre-processing for the dataset and a greedy algorithm for the selection of hyper-parameters. The superiority of the method was verified in both experiments and actual applications.

## 1. Introduction

**Figure 1:**
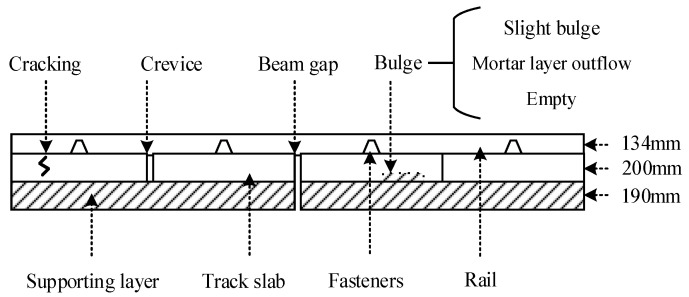
Typical defects in track detection.

Track defect detection: As a result of the high frequency and intensity of track operation in open air, track defects occur constantly. There are four typical defects in track detection: crevice, beam gap, cracking, and bulge, as shown in Figure 1. It should be noted that in the horizontal direction, we did not draw under the actual size. For actual track, the length of the track slab is 6450 mm. Bulges can be divided into multiple states according to their severity, such as slight bugles, mortar layer outflow, and empty [1]. Since the monolithic concrete structure of the track and the reinforcement are densely distributed, it is difficult to identify the defects inside. In traditional detection, in addition to the lack of real-time performance, the track detection vehicle has a long operating period and high deployment cost, and manual detection is restricted by the high error rate [2,3]. Therefore, approaches based on optical sensors have become the most remarkable methods in terms of deployment costs and real-time performance.

Optical sensors in track detection: Bao et al. [4] monitored the temperature and strain of joints by pulse-pre-pumped Brillouin optical time-domain analysis (PPP-BOTDA) with a single-mode fiber as a distributed sensor. Kang et al. [5] developed an FBG sensing system and graphical user interface (GUI) to monitor the wheel thickness changes in real time, which has been verified through the 1/6th sub-scale model test. Zhang et al. [6] proposed a track temperature prediction system based on FBG sensors and relevance vector regression theory, developed a real-time online monitoring system for railway track temperature, displacement, and strain, and deployed it as a Guangzhou–Shenzhen–Hong Kong high-speed railway track state warning system. Buggy et al. [7] monitored the condition of fishplates, stretcher poles, and switch blades using FBG strain sensors. However, due to broken bare fibers and low-strain-sensitivity jacketed fibers, the high cost and difficulty of deployment cannot be avoided [8]. In this case, in terms of deployment, DAS is considered the most suitable optical sensing system for track detection. However, DAS is mostly applied to detect train locations, speeds, and track incidents, but it has not yet been applied to multi-type minor defects detection [9,10,11]. Furthermore, the existing methods often provide a particularly determined output (so-called hard decision), which strongly depends on human knowledge, and the unique characteristics of the defects are required. Unfortunately, the unique characteristics of many defects have not been clearly defined.

Deep learning-based methods: As a result of the high-frequency sampling and low deployment cost, DAS is considered a data collection method that is perfectly suitable for various track detection technologies, especially deep learning methods. As a soft-decision method, deep learning provides a probability for each possible decision. An increasing number of researchers are focusing on deep learning for track detection. Based on deep learning, Wang et al. [12] pre-processed training data and defined the severity level to classify different severity levels for cracks in ballast-less tracks. ENSCO’s RIS (Railway imaging systems) has advanced high-resolution image acquisition systems and image processing algorithms, which can continuously detect fasteners during the day and night [13]. In [14], 2D images are transformed into 1D signals by Gabor filter, and then, the multiple signal classification (MUSIC) algorithm is used to detect the 1D signals, which can classify the signals produced by different track components. Yao et al. [15] used an artificial neural network (ANN) and a long short-term memory (LSTM) network to predict the frost heave deformation of a railway subgrade with four sections of data. Wei et al. [16] used Dense-SIFT, CNN, and R-CNN to detect defects in fasteners. Zheng et al. [17] developed a deep transfer learning (DTL) framework for rail surface crack detection using a limited volume of training images. Based on the YOLO V3 method, Wei et al. [18] proposed a fast detection model for exterior substances. Wang et al. [19] proposed a scheme for rail track state detection with a deep convolutional network as the core. Fan et al. [20] has shown how DAS system can be used for crack detection without the use of deep learning. They used vibration pulses to locate cracks on the track. However, many track defects, such as bulge, do not cause obvious vibration pulses, so it is difficult to detect them by Fan’s method. In addition, there are many kinds of track defects (event) that may lead to vibration pulses, such as switch, crack, and corrugation, and Fan’s method cannot distinguish them. Therefore, deep learning is a reasonable solution for multi-type defect detection.

Deep networks often achieve their strong performance through supervised learning, which requires labeled datasets. Therefore, the performance benefit conferred by the use of a larger dataset can come at a significant cost, because labeling data often requires human labor [21]. On the other hand, for track detection, there are many hidden defects that are difficult to be found or labeled, such as crack and bulge, which occur frequently during daytime operation and gradually disappear at night as the temperature drops. Therefore, we considered realizing track detection in a semi-supervised deep learning method. Semi-supervised learning (SSL) often achieves outstanding performance in the extremely scarce-label regime, with the efficient leveraging of unlabeled data [22,23,24]. In SSL models, decision boundaries are reinforced by unlabeled data, which enables the use of large, powerful models [25]. Owing to the fact that there are more unlabeled data than labeled data in actual conditions, the application of SSL in engineering has become a hot research field. Summarizing the previous work, the current DAS-based track defect detection system can be roughly divided into three categories: amplitude visualization, methods based on traditional machine learning, and methods based on deep learning. The existing deep learning-based methods mostly extract features from single-point vibration data and use MLP models to build classifiers. In this paper, we propose a track detection system that innovatively leverages semi-supervised deep learning based on image recognition. The innovation of this article is as follows:

(1) To increase the sample information density, we use multi-point amplitude rather than single-point;
(2) To alleviate the impact of the lack of high-frequency components caused by an insufficient sampling rate, we use amplitude rather than frequency features to train the model;
(3) We convert the data into images and classify the samples through a CNN network to achieve better convergence speed and capacity;
(4) We use the deep network to adaptively extract the sample features rather than manually extract them;
(5) We use semi-supervised learning to efficiently leverage unlabeled data to further improve the performance of the model.

Through the above-mentioned innovations, we successfully implemented a real-time track defect detection system and achieved superior performance, especially for multi-type minor defects. The structure of this paper is as follows. In the Section 2, we describe the deployment of the DAS and the distribution of defects on the experimental track. In the Section 3, we introduce the mechanism of SSL and several SSL models for comparison in the experiment. In the Section 4, we present a particular dataset pre-processing for our semi-supervised learning model, and we validate it in the Section 5. In the Section 6, we summarize our work and future research.

## 2. Sensor Deployment

**Figure 2:**
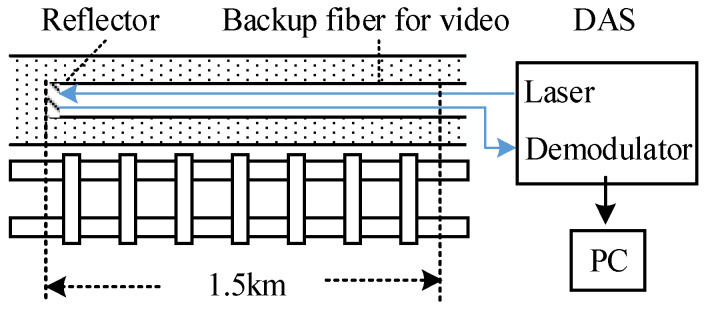
DAS deployment. The backup optical fiber for video is used as a sensor for DAS, which means that additional installation is not needed.

**Figure 3:**
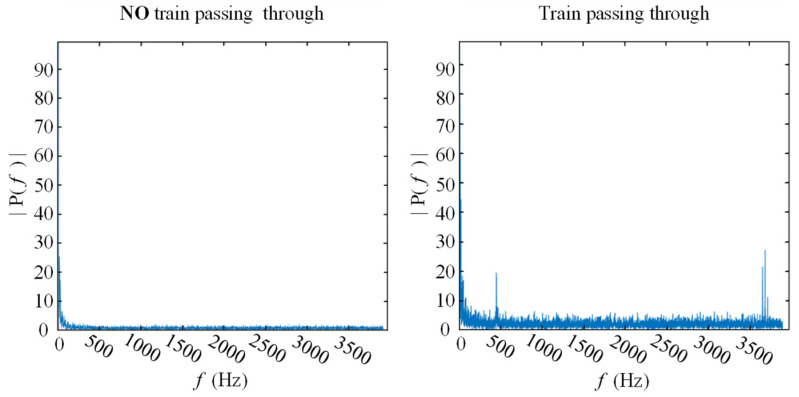
The spectrograms of the measured point with train passing through (**right**) and without train passing through (**left**).

Due to the security policy, experimental deployment along the railway track is not allowed. So, the existing backup fiber for video along the track is used as sensors, which means that additional installation is not needed. The fiber joint was connected to the DAS in the computer room. We deployed the commercial DAS with 5.8 m spatial resolution, measurement frequency range <5 kHz, and the fiber is G652D type. The DAS we used is an intensity-based DAS capable of only detecting vibrations. The vibration amplitude of the measurement points at each moment is buffered in the DAS in the form of a line of data; then, it is received and stored in a PC, as shown in Figure 2. The length of the optical path was measured to be 3000 m. By knocking around the fiber and observing the pulse position, we determined that the length of the optical path corresponding to the experimental track segment was approximately 1500 m, and the sampling rate is 2 kHz (2000 rows/s). The spectrograms of the measured points in the raw data are shown in Figure 3.

There are four typical defects to be recognized in our work including crevice, beam gap, cracking, and bulge, and in order to facilitate the analysis, two events causing peculiar vibrations serve as ‘defects’ for experimental object expansion, including switches and highway below. The distribution of defects is shown in Table 1. In actual conditions, events are difficult to locate accurately, so we roughly estimate it at 10 m intervals, which has been approved by maintainers. For ease of analysis, positions without events will be labeled ‘no-event’ in the experiment, but we did not list them in Table 1.

## 3. Data Representation

**Figure 4:**
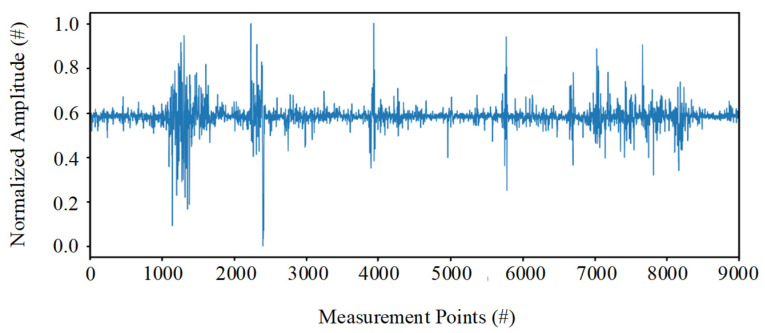
An example of normalized amplitude of the whole track at a certain moment.

Most of the existing methods are based on values, and our method focuses on the pattern behind them, which is called feature representation in deep learning methods. Fitting the actual data into deep learning models is one of the most important steps in deep learning. To make sure that the data values are comparable instances widely, Min–Max normalization is performed in order to prevent neuron output saturation or small values being ignored caused by excessive input absolute value:

(1) $$x^{\prime }=\frac{x-X_{min}}{X_{max}-X_{min}}$$

where $X_{min}$ and $X_{max}$ are the maximum and minimum of the entire dataset, respectively. An example of normalized data is shown in Figure 4.

Note that not all of the vibration data were added to the dataset. We filter the data by amplitude and then concatenate the fragments from different moments to ensure that the dataset is composed of vibrations caused by train passing. The train can be considered a scanner, and when the train passes through the entire experimental track, the vibration caused by the train will be recorded. To facilitate further descriptions, the $t_{0}$ row of the dataset can be considered a snapshot of the track at moment $t_{0}$.

Since data in the same row actually come from different moments and represent the vibration amplitudes of the entire track, we divide the data into spatial fragments in rows. In this way, the sampling rate requirement of the Nyquist criterion for DAS is avoided (only if $f_{sampling}>2\cdot f_{signal}$ can the integrated information be retained), because it is unnecessary to analyze the vibration modes of the measurement points in continuous periods in the frequency domain. Instead of detecting all of the measurement points, we only need to detect the fragments, thus reducing the computational cost.

**Figure 5:**
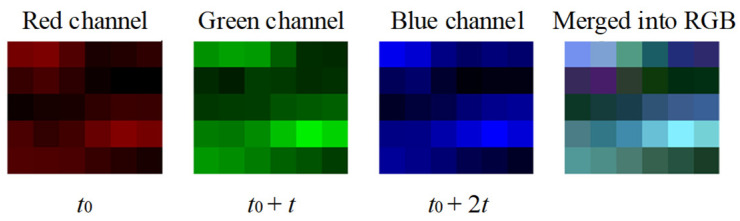
An example for merging the instances corresponding to the same fragment with a time interval *t* into one RGB image.

In our scheme, an instance can only describe the amplitude of a fragment at a certain moment, which is considered not comprehensive enough to represent the condition of the fragment. According to the mutual reasoning relationship among the instances at different moments, we merge the instances corresponding to the same fragment with a time interval t into a qualified instance in the form of an RGB image. An example of merging instances is shown in Figure 5. The time interval t is selected for validation as a hyper-parameter.

## 4. Semi-Supervised Deep Learning

**Figure 6:**
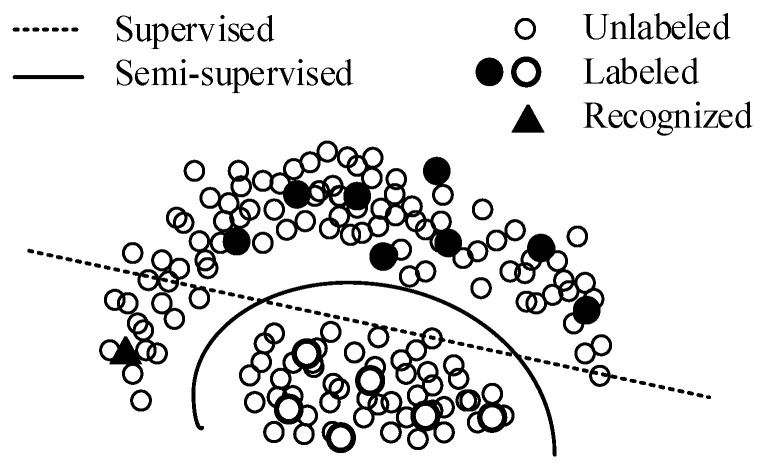
The decision boundary reinforcement by semi-supervised learning. The recognized instance will be class ‘hollow’ in supervised learning. However, the fact is that according to the distribution of the dataset, it should belong to class ‘solid’, which can be achieved in semi-supervised learning.

Semi-supervised deep learning (SSL) leverages unlabeled data to assist the training under four basic assumptions: smoothness assumption, low-density assumption, manifold assumption, and cluster assumption [26,27]. SLL reinforces the decision boundary according to the distribution of unlabeled data, as shown in Figure 6. Figure 6 is a two-dimensional schematic diagram, which abstractly describes the classification using the deep learning method. Solid circles and hollow circles represent the labeled samples in different classes respectively, smaller circles represent unlabeled samples, and the triangle is a sample belonging to the solid class. In the supervised learning method, the samples are represented in the form of vectors, and the model learns a decision boundary for dividing vector clusters (as shown by the dotted line in Figure 6). Under the decision boundary, the triangle sample will be determined to the hollow class. However, considering the unlabeled samples, even if they have no labels, we can still obtain a more reasonable decision boundary through their distribution (as shown by the solid line in Figure 6). Now, the triangle sample will be correctly determined to the solid class. Figure 6 shows how semi-supervised learning helps the model establish a more reasonable decision boundary leveraging the unlabeled samples.

SSL has been extensively studied by researchers, and various solid SSL models have been proposed. One commonly used class of SSL is based on the theory called consistency regularization [28], which is based on the intuition that the predictions of an instance and its perturbed version should be consistent for a qualified classifier. Therefore, to reinforce the decision boundary, we can minimize the divergence between the predictions of perturbed versions of the same unlabeled instance, such as the temporal ensemble (Pi model) [29], mean teachers [30], and UDA [31]. Another main approach to leverage unlabeled data is called ‘pseudo labeling’, where unlabeled data are given ‘guessed’ labels and join the labeled dataset for further training, such as Co-training [32] and Tri-training [32]. In addition, many holistic SSL strategies with superior performance have been proposed in recent years, such as Mix-match [33], Remix-match [34], and Fix-match [22]. Fix-match obtained state-of-the-art in the standard experimental setting described by Odena et al. [35].

### 4.1. Approaches of Data Augmentation for SSL

Most SSL strategies build loss functions with the help of data augmentation based on consistency regularization. Data augmentation in SSL can be divided into two types: weak and strong. Weak augmentation includes flip, shift, rotation, and scale, which can only produce slight distortion. On the contrary, strong augmentation can cause heavy distortion by crops, Gaussian blur, dropout, and so on. According to research in UDA, the SSL model can be significantly improved by suitable data augmentation [31]. Enlightened by this, the commonly used strong augmentations, such as Auto-Augment, Rand-Augment, and CT-augment, are dedicated to the selection of a set of transformations suiting the task better. CT-Augment outperforms the others in terms of computational cost and efficiency [24].

### 4.2. Loss Function and Pipeline

**Figure 7:**
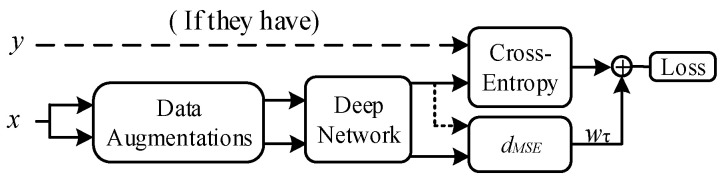
Pipeline of SSL based on consistency regularization.

Except for wrapper methods (such as Co-training, Tri-training, and Co-forest), there is always a loss term corresponding to the unlabeled data in the loss function in SSL. Consistency regularization methods build loss terms utilizing the predictions of two different perturbed versions of the same unlabeled data via the loss function:

(2) $$loss_{unlabeled}=w_{\tau }\cdot \overset{N}{\underset{i}{\sum }}d_{MES}\left[p_{m}\left(u_{i}^{\prime }\right),\;p_{m}\left(u_{i}^{\prime \prime }\right)\right]$$

where N is the amount of unlabeled data, $u_{i}^{\prime }$ and $u_{i}^{\prime \prime }$ are two different perturbed versions of unlabeled data $u_{i}$, $p_{m}$ is the prediction presented by the model, and $w_{\tau }$ is the weight of the unlabeled loss term. We need to change $w_{\tau }$ over the training because unlabeled data cause too much disturbance in the early stages. The pipeline of the SSL based on consistency regularization is shown in Figure 7.

**Figure 8:**
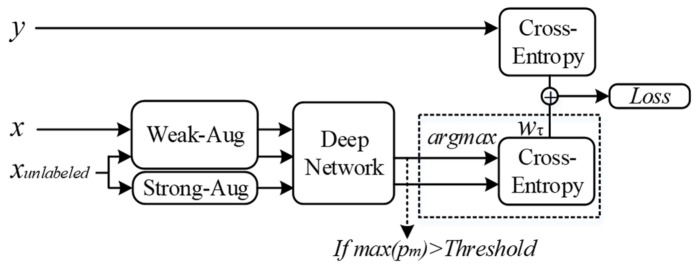
Pipeline of Fix-match.

In pseudo-labeling methods, unlabeled data will be given a pseudo label if the model is confident enough in the prediction. In each round of iteration, the cross-entropy between the prediction of unlabeled data and their pseudo labels (if they have) will be made and join the global loss via the loss function:

(3) $$loss_{unlabeled}=w_{\tau }\cdot \overset{N}{\underset{i}{\sum }}IF(\max\left(p_{m}\left(\alpha \left(u_{i}\right)\right)\right)>\beta )H\left[y_{m}\left(\alpha (u_{i})\right),\;p_{m}\left(A(u_{i})\right)\right]$$

where β is the threshold that determines whether the model is confident enough. $y_{m}\left(\alpha (u_{i})\right)$ is the pseudo label of unlabeled data $u_{i}$ under weak augmentation α, and A is a strong augmentation. The pipeline of the Fix-match is shown in Figure 8.

In order to select the most suitable SSL model for track detection, we will use the SSL models as a hyper-parameter in validation, including UDA, Tri-training, and Fix-match.

## 5. Experiment

### 5.1. Experiment Setting

The raw dataset contains 1869 rows of vibration data, and each row of data contains 9000 measurement points. A row of data corresponds to the vibration caused by a train passing through the track. Except for conventional data processing including de-noise, normalization, we do the instance merging proposed in Section 4 and divide the entire experimental track into 300 fragments with a 5 m interval, so the spatial accuracy of our research is as follows:

(4) $$\pm \frac{1500/300}{2}=\pm 2.5\;\left(m\right).$$

Each row of data was divided into 300 instances, and each instance contained 9000/300=30 columns. We labeled each instance according to the defect distribution described in Table 1. The overall dataset is presented in Table 2.

It can be seen from Table 2 that the imbalance problem of the overall dataset is quite serious, especially class ‘no-event’, which is ten times larger than the minority class. Therefore, we process the dataset to the size described in Table 2 by one of the well-performing balance methods, which will be selected in the validation as a hyper-parameter. Twenty percent of the overall dataset was randomly selected to constitute the testing set, and the remaining instances are used as the training set. To obtain unlabeled instances, we define a parameter μ:

(5) $$\mu =N_{unlabeled}/(N_{labeled}+N_{unlabeled})$$

where N is the total number of training sets and $N_{unlabeled}$ is the number of unlabeled instances that we need. In the validation, each label has a probability μ to be removed. For our experiment, μ is set to 50%. The training set is presented in Table 3.

Table 4 identifies a set of hyper-parameter ranges between the maximum and minimum to optimize the use of BO in deep learning networks based on the survey [36]. In addition to these hyper-parameters, there are four more hyper-parameters defined in our research, including the structure of the deep network, data balance method, SSL model, and the time interval t defined in Section 4.

### 5.2. Validation

The four structural hyper-parameters for our research, which are shown in Table 5, are selected by the greedy algorithm. SMOTE-TL, S-ENN, Border-1, MWMOTE, and Safe-level are the most commonly used oversampling methods, in which artificial samples are generated by linear combinations of existing samples. The difference between them lies in the samples selected for linear combination, which is related to the dataset. Therefore, we need to choose a data balance method suitable for our dataset through experiments. The time interval *t* is discrete and is just a virtual concept representing the number of instances between the instances used to merge. Since the train is considered a scanner and the fragments from different rows are scanned by different trains, *t* represents the running interval of the trains. Approximately 54 trains pass through the experimental track every day, so we set the upper limit of the value range of *t* to 54, which means that an instance can contain the amplitude data belonging to three moments of three days occurring on the same fragment at most. When *t* is 0, an instance can only contain the amplitude data belonging to the three trains passing continuously. There is a reasonable intuition that containing data from different moments on different days enables a qualitative description of the vibration mode of a fragment. The vibration mode of a certain fragment at different moments in different days should be different. Therefore, we empirically initialize *t* to 36.

#### 5.2.1. Validation on Network Structure and Data Balance Method

Since both are strongly related to the data distribution, the network structures and balance methods are selected in combinations. The evaluation metrics we used here are AUC, F1, and g-mean, which are the most used evaluation metrics for classifiers in the data imbalance situation. We conducted experiments on all combinations of network structures and balance methods. The details of the results are presented in Table 6, where the results of the best combinations under AUC, F1, and g-mean are in bold. The two best combinations, VGG-16 with S-ENN and ResNet with MWMOTE, will be used for further validation.

#### 5.2.2. Validation on SSL Model

**Figure 9:**
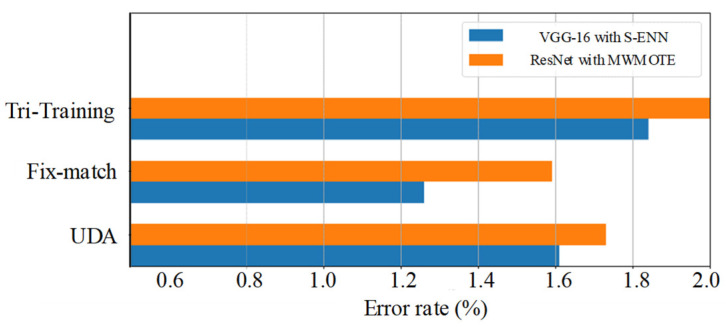
Error rates of different SSL models under different network structures.

Strongly related to the network structures, the SSL model will be selected under the two combinations selected, including VGG-16 with S-ENN and ResNet with MWMOTE. The error rates of the different combinations are shown in Figure 9. For our task, it can be seen that the combination of Fix-match, VGG-16, and S-ENN is the best.

#### 5.2.3. Validation on *t*

**Figure 10:**
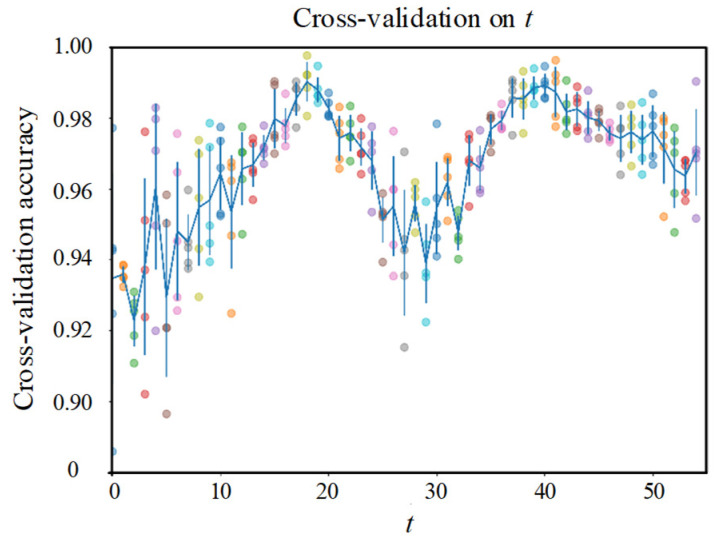
Five-fold cross-validation on *t*.

**Figure 11:**
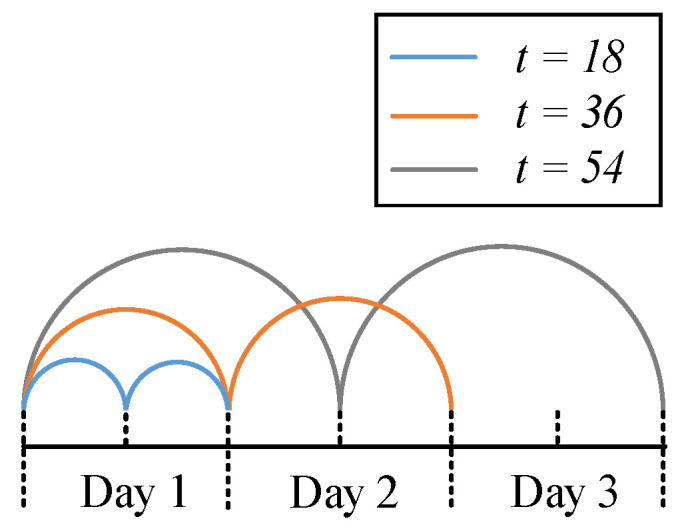
A description of how hyper-parameter *t* works.

We performed five-fold cross-validation on t, and the results are shown in Figure 10. There are two peaks at 17 and 39 in the box check of *t*, and the curve is approximately periodic with period 18. An instance is merged from three different trains passing through, and there are approximately 54 trains passing through a day. Therefore, the peak at 17 indicates that when the information contained in an instance is dispersed as much as possible in a day, the contribution of this instance is the largest, because the different vibration modes of the fragment at different times in a day are considered, as shown in Figure 11. In addition, considering the standard deviation, as the interval of the fragments contained in the instances becomes larger, the performance of the system becomes more stable.

### 5.3. Comparison

**Figure 12:**
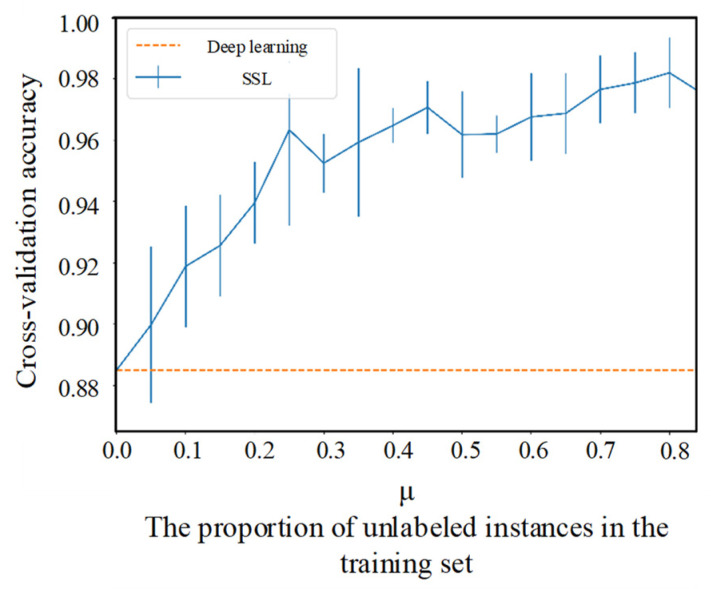
Comparison of SSL and deep learning method trained by the same amount of labeled data. The μ is the proportion of unlabeled instances in the training set defined in the Equation (5).

According to the pipelines of SSL models, when there are no unlabeled instances used, the SSL model may degenerate into a deep learning model. Therefore, we compare the SSL and deep learning methods trained using the same amount of labeled data by fixing the number of labeled instances and changing μ. The amount of labeled data in each class was fixed at 500, and the range of μ was [0, 0.84]. The results are presented in Figure 12.

### 5.4. Testing

The optimal hyper-parameters are selected according to the validation, as shown in Table 7. The result of testing is presented in the form of a confusion matrix, and the element $e_{ij}$ is the amount of i predicted as *j*. The precision and recall of multi-classification are defined by

(6) $${\mathrm{Precision}}_{i}=\left(\sum^{}i\;\mathrm{prediceted}\;\mathrm{as}\;i\right)/\left(\sum^{}\mathrm{instance}\;\mathrm{predicted}\;\mathrm{as}\;i\right)$$

(7) $${\mathrm{Recall}}_{i}=\left(\sum^{}i\;\mathrm{prediceted}\;\mathrm{as}\;i\right)/\left(\sum^{}i\right).$$

**Figure 13:**
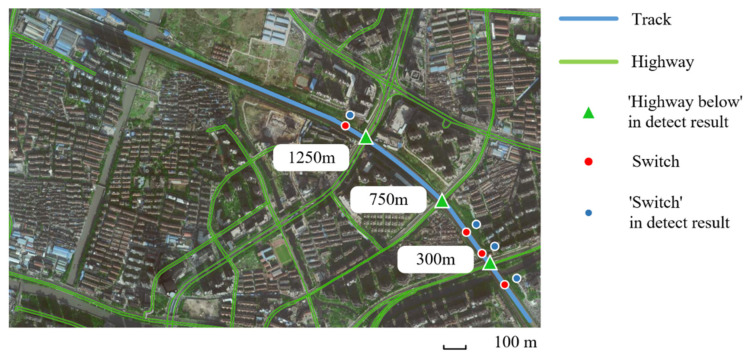
Visualization for detection on actual data.

Precision and recall are the most commonly used evaluation metrics for the classification. The superiority of our method is shown in Table 8, and a visualization for detection is shown in Figure 13. It should be noted that in the real world, amplitude variation may be caused by a variety of situations, such as changes in the state of the train, different exposed states of the cable, and unexpected changes in the natural environment. The peaks in Figure 13 are mainly caused by the above unexpected situation and have less correlation with track defects in which we are interested.

For safety considerations, accuracy is not always the most important metric for real-time track detection. A high recall rate of defects and a high precision rate of no-defect are always pursued to avoid defect omissions. The results of a two-class actual dataset including ‘defect’ and ‘no-defect’ are shown in Table 9. It can be seen that most of the defects are found (recall of defects = 0.9938), and the omissions are rare (precision of no-defects = 0.9938), which can superiorly meet the safety requirements.

Compared with the relevant work [19] (as shown in Table 10), our method is end-to-end, which is conducive to a more convenient model adjustment and faster operation speed. With the efficient use of unlabeled data and greater sample information density, our model achieves higher accuracy in more complex tasks.

## 6. Discussion

In this paper, we propose a track detection method that innovatively leverages semi-supervised deep learning based on image recognition, with a particular dataset pre-processing and a greedy algorithm for the selection of hyper-parameters. The accuracy reached 97.91%, which is satisfactory. In this section, we discuss some details of our research and point out our limitations, and the ideas for further research are proposed.

**Figure 14:**
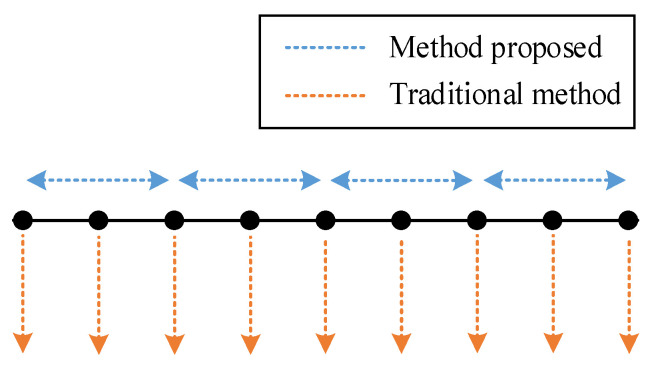
The difference between traditional methods and method proposed.

Firstly, there is a trade-off between the traditional deep learning-based methods and our method. We achieved a low computational cost and low sampling frequency requirement in detection with the cost of spatial accuracy, as shown in Figure 14. However, traditional methods perform better in terms of spatial accuracy. Moreover, according to the validation results on *t*, time and environment can make a difference in the vibration modes of the track, which is consistent with the intuition of the researchers. This may bring some new solutions on how to expand (or augment) datasets when labeled data are limited in engineering situations. Using data from different times and environments may be a type of data augmentation analogous to flip and shift for image recognition. In addition, an outperformed classifier is not always a good solution for specific tasks. Only when meeting the different biases of each class required in the actual project can a classifier be applied, which is an important factor in the application of deep learning. For DAS, higher spatial resolution may lead to higher prices. However, in our method, higher spatial resolution means that each device can cover a longer distance. Therefore, using expensive equipment is counter-intuitively a more economical approach. In addition, due to the bottleneck of transmission rate, embedding the defect detection system into the DAS is a low-cost method to improve the actual sampling rate and detection accuracy of the system.

The main drawback of our method is that our method cannot evaluate the severity of defects and their evolution. We used deep learning models, so in fact, we did not actually model the mechanism of defects. To evaluate the severity and the evolution, we must use a dataset with severity labels. It is difficult to obtain such a dataset because it requires professionals to manually label the severity of the defects and requires high labor cost. In addition, there are still some limitations of our research:

(1) Since the defects are rare on the railways in operation, and the tracks that can be used for research are very limited due to the security policy, there are not enough defects that can be used in our research to prove that all kinds of defects can be found by the proposed method. Considering the base assumption of SSL mentioned in Section 3, in other defects, there may not be a special density area for classification.
(2) Under special conditions, such as in a tunnel full of echoes, vibration may be distorted owing to the influence of complex environmental noise, leading to classification errors. In addition, it is almost impossible to detect under a large amount of environmental noise in a data-driven manner. Therefore, it is necessary to preprocess the data according to the environment.
(3) The vibration modes of the defects may change with environment, and automatic online learning may lead to errors being inherited and amplified. Therefore, a traditional track detection method is necessary for updating the proposed method.
(4) According to the security requirements of high-speed railways, a detection system based on black-box models cannot be fully trusted. Thus, it can only be used as a supplement in real-time detections.
(5) In fact, our method is a classification task rather than a recognition task, and the number of categories is set in advance. Therefore, we need all types of defects to be marked before the training. However, it is difficult to guarantee that all defects are marked in a general situation. In order to solve this problem, we can add an additional ‘unknown’ category and classify samples that are not similar to existing training samples into this category.

There are two main research directions in this area. The first is the interpretability of the black-box models. Although we have explained the safety-related cost sensitivity classification in Section 5.4, the black-box models based on deep learning still cannot be fully trusted in safety-related areas. We should try to combine deep learning with traditional machine learning, such as decision trees, and try to use white-box models to explain the intermediate steps of the black-box models. Secondly, data representation is another direction of concern. It is the key to deep learning applications in engineering tasks that transform engineering problems into general deep learning problems. Finding a more suitable data representation to fit actual data into the existing deep learning model will be one of our main research topics in the future.

## Figures and Tables

**Table 1:** Event distribution without ‘no-event’. (Position of 0 m: the end near the computer room; Position of 1500 m: the other end).

Event	Location
Crevice	120 m, 790 m, 830 m, 1010 m, 1270 m
Beam Gap	400 m, 500 m, 600 m, 700 m, 800 m, 900 m, 1000 m, 1100 m, 1200 m, 1300 m, 1400 m
Cracking	100 m, 480 m, 650 m, 1050 m
Bulge	420 m, 560 m, 730 m, 1030 m, 1420 m
Switches	200 m, 350 m, 450 m, 1350 m
Highway Below	300 m, 750 m, 1250 m

**Table 2:** (**a**). Overall dataset obtained by data segmentation. (**b**). Balanced dataset obtained by oversampling from the original overall dataset.

**(a)**			
**Size**	**Channels**	**Event**	**Amount**
5 × 6	3	Crevice	3115
		Beam gap	6853
		Cracking	2492
		Bulge	3115
		Switches	2492
		Highway below	1869
		No event	166,964
**(b)**			
**Size**	**Channels**	**Event**	**Amount**
5 × 6	3	Crevice	6853
		Beam gap	6853
		Cracking	6853
		Bulge	6853
		Switches	6853
		Highway below	6853
		No event	6853

**Table 3:** Training set for classification model training generated from the balanced overall dataset.

Size	Channels	Event	Amount
5 × 6	3	Crevice	2708
		Beam gap	2788
		Cracking	2735
		Bulge	2558
		Switches	2776
		Highway below	2736
		No event	2716
		Unlabeled	19,359

**Table 4:** Optimized hyper-parameters for optimizers and their search ranges. SGDM, Rmsprop, Adam are gradient-based model optimizers. Since different models and datasets require different parameter update rules, it is necessary to select the optimal optimizer. The optimal parameter combination for optimizers also needs to be selected by random search in a certain range, including the initial learning rate, momentum, SGDF, GDF, and L2 regularization.

Hyper-Parameters	SGDM	Rmsprop	Adam
Initial Learning Rate	[1×10−2, 1]	[1×10−3, 1]	[1×10−3, 1]
Momentum	[0.3, 0.95]	N/A	N/A
SGDF	N/A	[0, 1]	N/A
GDF	N/A	N/A	[0, 1]
$L_{2}\;$Regularization	[1×10−9, 1×10−2]	[1×10−9, 1×10−2]	[1×10−9, 1×10−2]

**Table 5:** Structural hyper-parameters for our monitor system.

Hyper-Parameters	Options
Structure of deep learning network	VGG-16, ResNet, Inception V3, AlexNet, Mobilenet V3, LeNet
Data balance method	SMOTE-TL, S-ENN, Border-1, MWMOTE, Safe-level
SSL model	Fix-match, Tri-training, UDA
Time interval (*t*)	[0, 54]

**Table 6:** Results of various combination of deep learning network structures and data balance methods.

Measure	Structure of Deep Learning Network	Data Balance Method				
		SMOTE-TL	S-ENN	Border-1	MWMOTE	Safe-Level
AUC	VGG-16	0.9832	**0.9862**	0.9740	0.9756	0.9736
	ResNet	0.9763	0.9704	0.9632	0.9810	0.9803
	Inception V3	0.9620	0.9734	0.9680	0.9649	0.9784
	AlexNet	0.9745	0.9720	0.9820	0.9816	0.9803
	Mobilenet V3	0.9609	0.9832	0.9783	0.9734	0.9760
	LeNet	0.9753	0.9767	0.9734	0.9809	0.9719
$F_{1}$	VGG-16	0.7095	**0.7103**	0.6943	0.7081	0.7079
	ResNet	0.7057	0.6823	0.6972	0.7127	0.7084
	Inception V3	0.5970	0.6490	0.5408	0.6701	0.6978
	AlexNet	0.6437	0.6824	0.6920	0.6845	0.6450
	Mobilenet V3	0.6920	0.6538	0.6823	0.5970	0.6784
	LeNet	0.7009	0.6903	0.6574	0.6739	0.6273
g-mean	VGG-16	0.9708	0.9719	0.9683	0.9607	0.9608
	ResNet	0.9692	0.9717	0.9631	**0.9729**	0.9687
	Inception V3	0.9582	0.9703	0.9602	0.9636	0.9705
	AlexNet	0.9680	0.9648	0.9538	0.9670	0.9643
	Mobilenet V3	0.9605	0.9567	0.9629	0.9534	0.9617
	LeNet	0.9601	0.9584	0.9597	0.9607	0.9658

**Table 7:** Optimal hyper-parameters selected in the validation.

Optimal Hyper-Parameters	
Structure of deep learning network	VGG-16
Data balance method	S-ENN
SSL model	Fix-match
Time interval (*t*)	17

**Table 8:** Confusion matrix for testing.

Actual	Crevice	Beam Gap	Cracking	Bulge	Switches	Highway Below	No Event	Total
Predict								
Crevice	1337	6	5	1	6	6	1	1362
Beam gap	6	1334	8	3	5	5	5	1366
Cracking	3	6	1343	5	7	2	2	1368
Bulge	7	7	6	1349	8	5	4	1386
Switches	5	1	1	4	1333	4	1	1349
Highway	8	9	5	5	5	1342	5	1379
No-event	4	7	2	3	6	6	1352	1380
Total	1370	1370	1370	1370	1370	1370	1370	
Precision	0.982	0.977	0.982	0.973	0.988	0.973	0.980	
Recall	0.976	0.974	0.980	0.985	0.973	0.980	0.989	
Accuracy	0.9791							

**Table 9:** Confusion matrix for classification of ‘defect’ and ‘no-defects’.

Predict/Actual	Defects	No-Defect	Total
Defects	8919	56	8975
No-defects	21	1004	1025
Total	8940	1060	\
Precision	0.9938	0.9795	\
Recall	0.9977	0.9472	\

**Table 10:** Comparison with previous work.

	Use of Unlabeled Data	Sample Structure	Defect Types	Mode	Acc
[12]	No	Image (Three channel)	1	End-to-end	93.00%
[16]	No	Image (Three channel)	1	End-to-end	97.14%
[17]	No	Image (Three channel)	1	End-to-end	92.00%
[19]	No	Three-point (channel)	4	Two-stage	94.98%
Ours	Yes	Multi-point (channel)	6	End-to-end	97.91%
